# Clinical feasibility evaluation of a digital workflow of prosthetically oriented onlay bone grafting for horizontal alveolar augmentation: a prospective pilot study

**DOI:** 10.1186/s12903-023-03556-0

**Published:** 2023-10-30

**Authors:** Yiman Tang, Shuyong Zhai, Huajie Yu, Lixin Qiu

**Affiliations:** 1 4Th Division, Biomaterials and Digital Medical Devices & Beijing Key Laboratory of Digital Stomatology & NHC Research Center of Engineering and Technology for Computerized Dentistry, Peking University School and Hospital of Stomatology & National Center for Stomatology & National Clinical Research Center for Oral Diseases & National Engineering Research Center of Oral, Beijing, 100081 People’s Republic of China; 2 Dental Digital & Esthetics Laboratory, Beijing Shengzhuo Dental Corporation, Beijing, People’s Republic of China

**Keywords:** Digital workflow, Horizontal ridge augmentation, Onlay bone grafting, Dental implants

## Abstract

**Background:**

Onlay bone grafting is considered highly reliable for reconstructing severe horizontal bone defects. A critical problem is how to achieve precise position of the bone block to control alveolar ridge dimensions. This research aims to establish a digital workflow for prosthetically oriented onlay bone grafting and evaluate its accuracy and efficiency.

**Methods:**

This prospective pilot study investigated eight patients who required implant restoration in the esthetic area with horizontal alveolar bone defects. The workflow includes preoperative virtual planning, design and manufacture of patient-specific templates, bone grafting surgery, and implant insertion. Primary outcomes were graft accuracy, defined by root mean square estimate (RMSE) values between preoperatively designed and actual implanted outer contours of bone blocks. Secondary outcomes were bone graft and implant success rates. Besides, the surgeons used the visual analog scale (VAS) to rate the intuitiveness, ease of understanding, and helpfulness of the workflow.

**Results:**

No bone grafts or implants failed in any of the eight patients, resulting in a 100% success rate. The RMSE values between the preoperative design and the implanted outer contour of bone blocks were 0.41 ± 0.15 mm. The digital approach showed advantages in intuitiveness (9.3 ± 0.5), understanding (9.0 ± 0.5), and helpfulness (8.4 ± 1.1) according to surgeons' VAS scores.

**Conclusions:**

A digital workflow provided encouraging results, in terms of accuracy and efficacy, for horizontal bone augmentation.

**Trial registration:**

This study was registered in the National Clinical Trials Registry in 16/02/2023 under the identification number ChiCTR2300068361.

## Background

Severe periodontitis, trauma, and long-term edentulism can cause significant resorptive alterations of the alveolar ridge, leading to severe bone defects in the esthetic zone [1]. Poor implant position increases the incidence of complications such as marginal bone loss and peri-implantitis and requires frequent maintenance. In addition, sufficient bone tissue is a prerequisite for ideal implant placement and long-term esthetic results [2]. The use of autogenous bone is the key factor for reconstructing severe bone defects because of its excellent osteoconductive, osteoinductive and osteogenic properties, both in onlay/inlay block grafts and in guided bone regeneration (GBR) [3]. Benic and Hämmerle suggested that a staged approach with onlay bone grafting is the most commonly used technique for class IV and V bone defects because of its reliability in maintaining stable bone height and width of reconstructed bone defects [4].

Emerging digital technologies and innovations have deeply changed the surgical procedures for different techniques of bone augmentation, such as titanium meshes [5–7], reinforced PTFE membranes [8], maxillary sinus lift [9–11], split bone blocks [12], and onlay bone blocks [13–15]. Figliuzzi first reported custom-made, computer-aided design/computer-aided manufacturing (CAD/CAM) hydroxyapatite scaffolds to augment posterior mandibular bone [16]. Misch reported the fabrication of a replica of the target bone block as a template for harvesting and placing autogenous bone grafts, ensuring adequate volume and proper positioning of the block grafts to reconstruct alveolar defects [17]. Pham Dang et al. used digital techniques to print a 3D model of the recipient region, which was used to trim the bone block in vitro to improve the match between the graft and the recipient region [18]. However, few workflows are available to simulate and guide the entire treatment process.

The surgical procedure of onlay bone grafting remains a challenging issue for surgeons. While the bone harvest technique is relatively well-developed, the positioning and fixation of the bone block in the recipient area rely solely on the surgeon's personal experience in conventional procedures [19, 20]. The lack of a guide for bone grafting may lead to deviated bone augmentation effects, undesirable implant positions, and unpredictable aesthetic outcomes. We herein presented a novel digital workflow for fabricating patient-specific CAD-CAM titanium templates to realize more efficient and predictable onlay bone grafting.

The primary aim of this pilot study was to evaluate the accuracy of CAD/CAM surgical templates for positioning autogenous bone grafts for further implant placement. The secondary aim of the study was to evaluate the surgeon-reported and patient-reported outcomes of the digital workflow. In addition, the inherent advantages and disadvantages of this approach were discussed.

## Methods

### Study design and recruitment criteria

The present study was designed as a prospective pilot study. The study was authorized by the Ethics Committee of Peking University School and Hospital of Stomatology (approval number PKUSSIRB-201840180) and performed under the guidelines in the Declaration of Helsinki (World Medical, 2013). The inclusion criteria were as follows: (1) patients who had single or multiple missing anterior teeth that required implant restoration; (2) patients with a horizontal bone width less than 4 mm, which is insufficient for accommodating a standard size implant; (3) patients who were at least 18 years old and can cooperate with follow-up visits. Patients with contraindications, such as local or systemic diseases, were excluded. All patients were treated in the 4th Division of Peking University School and Hospital of Stomatology. Eight patients (3 males and 5 females) who came to the clinic from October 2019 to October 2022 were recruited for this research.

### Digital workflow protocol

**Fig. 1:**
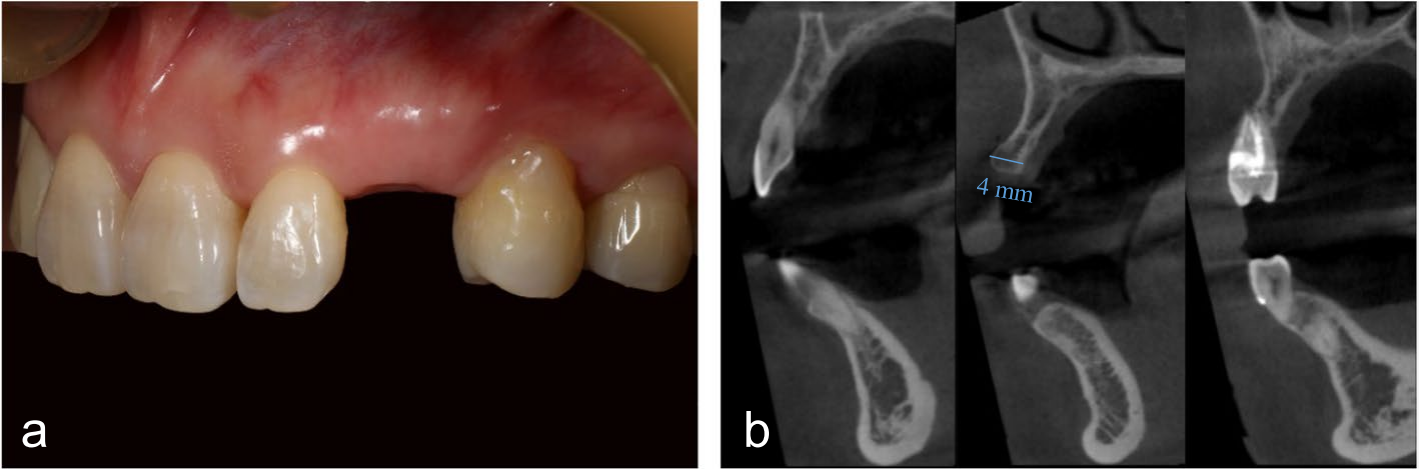
Preoperative examination. **a** Intraoral photograph; **b** severe horizontal bone defect revealed on CBCT

The complete prosthetically oriented onlay bone grafting procedure includes data acquisition, virtual planning, design and manufacture of surgical templates, and bone augmentation surgery. The specific process was presented as an example of a case. The patient had a missing maxillary left canine and required implant restoration (Fig. 1a). CBCT images showed a horizontal bone defect (Fig. 1b).

#### P0 – virtual planning and digital workflow

Digital information from the patient was collected, including the intraoral dentition model in the standard tessellation language (STL) file format by using a scanner (TRIOS; 3Shape) and cone beam computed tomography (CBCT) imaging in the Digital Imaging and Communications in Medicine (DICOM) file format. Threshold segmentation was performed in the DICOM file to obtain the 3D reconstruction of the jaw model in STL file format.

**Fig. 2:**
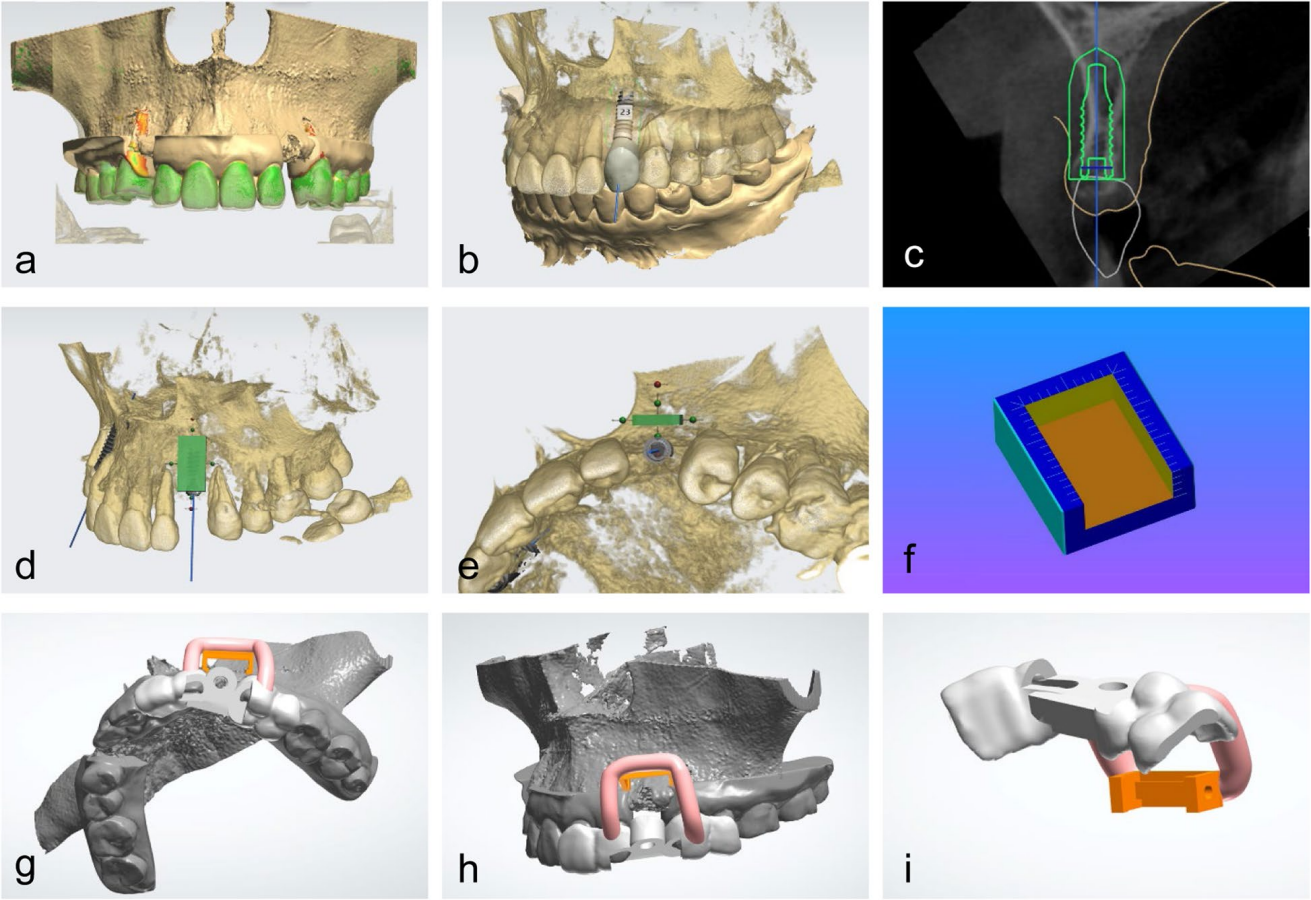
Design of definitive prosthesis, implant, bone block and template. **a** Dentition scan model superimposed onto 3-dimensional reconstructed maxilla; **b** planned definitive prosthesis and implant; **c** planned prosthesis (white) and implant position (green) on CBCT; **d**-**e** planned position of the bone block graft (green); **f** designed bone trim template; **g**-**i** designed bone graft template, including tooth-supported base template (white), clamp (orange) and rod-like attachment (pink)

The dentition model was superimposed onto the jaw model in the 3Shape software using the corresponding characteristic points on the crown of the tooth for registration, thus obtaining a merged digital model containing dentition and jaw bone information (Fig. 2a). The final prosthesis with virtual diagnostic waxing was designed on the merged digital model (Fig. 2b). The ideal implant position was planned in a prosthetically oriented manner in the 3Shape implant planning module (Fig. 2c). Then the 3D position of the bone block was designed based on the bone volume requirements around the implant and esthetic requirements of the bone arch profile. The coronal side of the bone block was located 3 mm below the ideal gingival margin, and the apical side reached at least 2/3 of the implant length (Fig. 2d). The thickness of the bone block was designed to ensure that at least 2 mm bone mass remained around the implant (Fig. 2e).

A universal trim template was designed to measure the length, width and height of the bone block (Fig. 2f). Based on the determined contour and boundaries of the bone block, a clamp that fits the shape and location of the bone block was generated. A tooth-supported base template was developed on 2–3 adjacent teeth with seating verification windows. The intermediate holes which were used to guide the implant placement were incorporated into the base template. A rod-like attachment structure was created to connect the clamp with the tooth-supported base template (Fig. 2g-i).

#### T0 – bone augmentation surgery

**Fig. 3:**
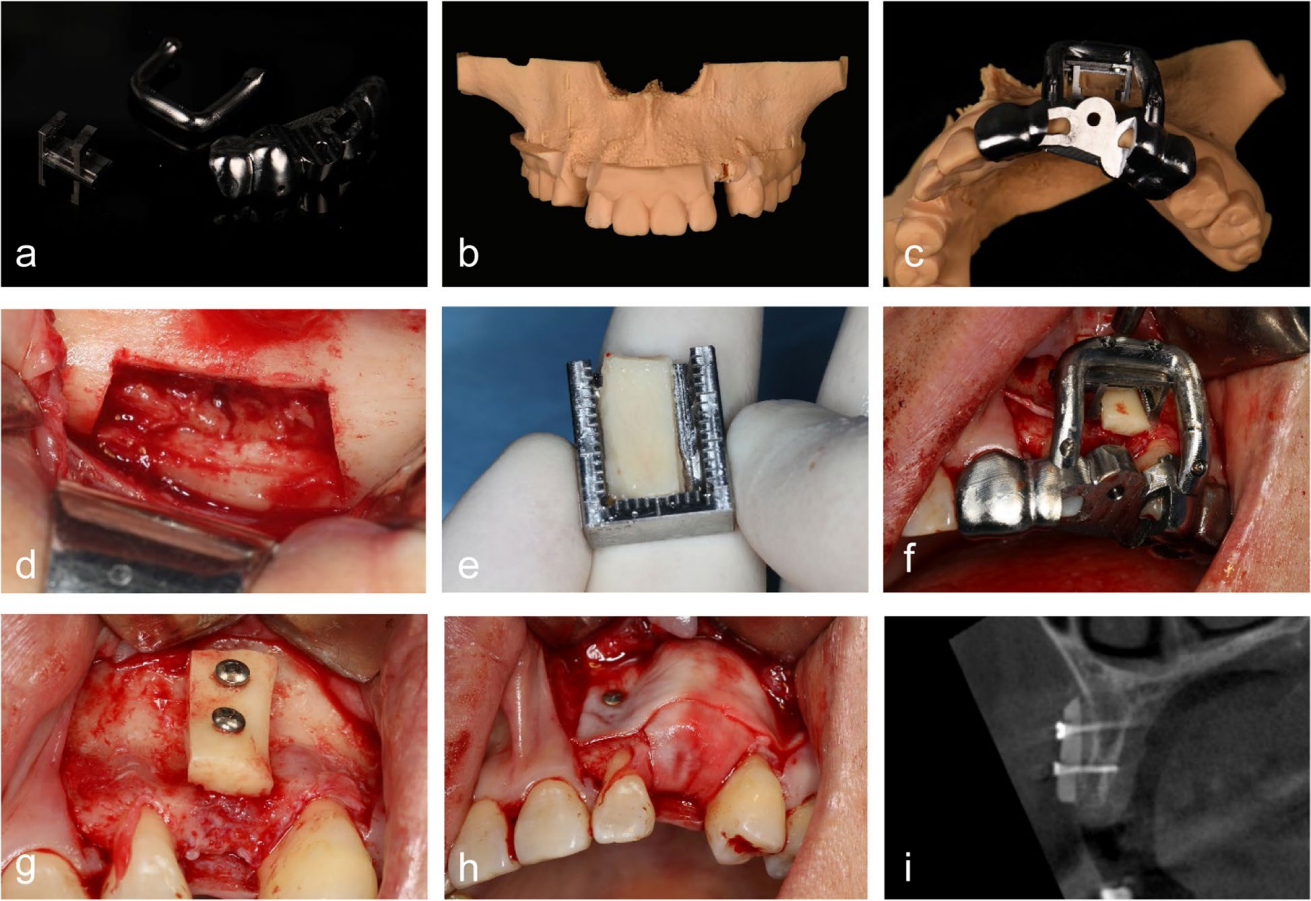
Onlay bone graft surgery. **a** Three parts of the graft template milled with titanium; **b** printed diagnostic cast; **c** template trying in diagnostic cast; **d** bone block extraction; **e** trimming of harvested bone block placed in trim template; **f** graft template positioned to complete drilling process; **g** bone block fixed with 2 screws; **h** the recipient area covered with resorbable collagen membrane; **i** postoperative CBCT imaging

Before surgery, the template was fabricated by using a 5-axis machining center (308S2, Willemin-Macodel SA) and titanium disk (Adentatec GmbH) (Fig. 3a). The diagnostic cast was printed with photopolymerizing resin by using a 3D printer (Shining 3D) (Fig. 3b). Different parts of the graft template were assembled by connecting screws, and the template was positioned on the diagnostic cast to check the fit (Fig. 3c).

The patients received prophylactic antibiotic treatment with 500 mg Cefuroxime Axetil tablets or 300 mg Roxithromycin capsules if allergic 1 h before the procedure. After sterilization and anesthesia of the surgical site, a horizontal incision was made on the alveolar ridge of the recipient area, and a vertical incision was made at the distal angle of adjacent teeth. The full-thickness mucoperiosteal flap was raised to expose the bone. The autogenous bone block was taken from the external oblique area of the mandible using a fissure bur. Then the bone block was placed in the trim template and trimmed with round burs to the planned dimension under the guidance of the template (Fig. 3d). After placing the bone block into the clamp of the template, the template was positioned intraorally in the recipient area. The connecting screws were locked to ensure complete seating (Fig. 3e). The holes were drilled in the bone, and the bone block was fixed to the recipient area with titanium screws (Fig. 3f). The gap was filled around the bone block with bone substitutes (Bio-Oss; Geistlich Pharma AG, Switzerland). Subsequently, the recipient area was covered with a resorbable collagen membrane (Bio-Guide; Geistlich Pharma AG, Switzerland) (Fig. 3g). Patients received CBCT examination immediately after surgery (Fig. 3h, i).

After surgery, patients were instructed to take 250 mg Cefuroxime Axetil tablets twice daily or 150 mg Roxithromycin capsules for allergic patients twice daily for 7 days to prevent infection. Additionally, ibuprofen extended-release capsules were recommended for pain relief. Patients were instructed to use 0.12% chlorhexidine mouthwash for 7 days postoperatively to control chemical plaque.

#### T1 – implant placement

**Fig. 4:**
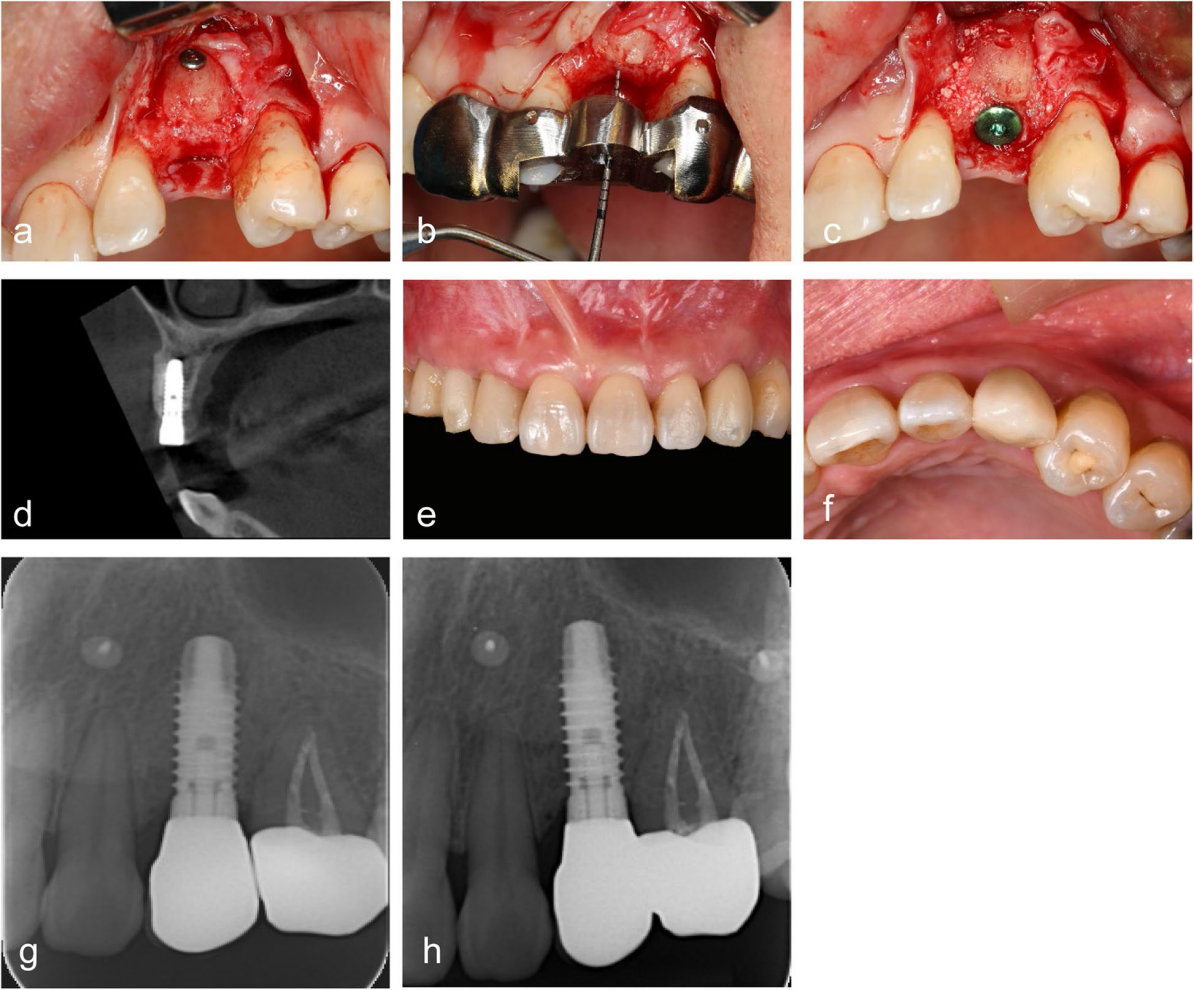
Implant placement and prosthesis loading. **a** Bone blocks with good osteogenic results after six months; **b** socket preparation following guidance of tooth-supported base template;** c** implant placement; **d** postoperative CBCT of bone block and implant;** e**–**f** screw-retained prosthesis; **g** X-ray of implant after final restoration; **h** X-ray of implant after 1 year of loading

After six months, upon good osteogenesis of the bone block, the screws that were used to stabilize the block were identified and removed (Fig. 4a). The implant socket was prepared under the guidance of a tooth-supported base template (Fig. 4b). An implant (Thommen Medical AG, Switzerland) was inserted according to preoperative virtual design (Fig. 4c, d).

#### T2 – functional loading (baseline)

After three months, an implant-supported prosthesis was delivered (Fig. 4e, f).

#### T3 – 1 year (follow-up)

Patients were asked to follow up after one year to observe and record the clinical condition of the implants and prostheses (Fig. 4g, h).

### Outcome evaluations

#### Graft accuracy

**Fig. 5:**
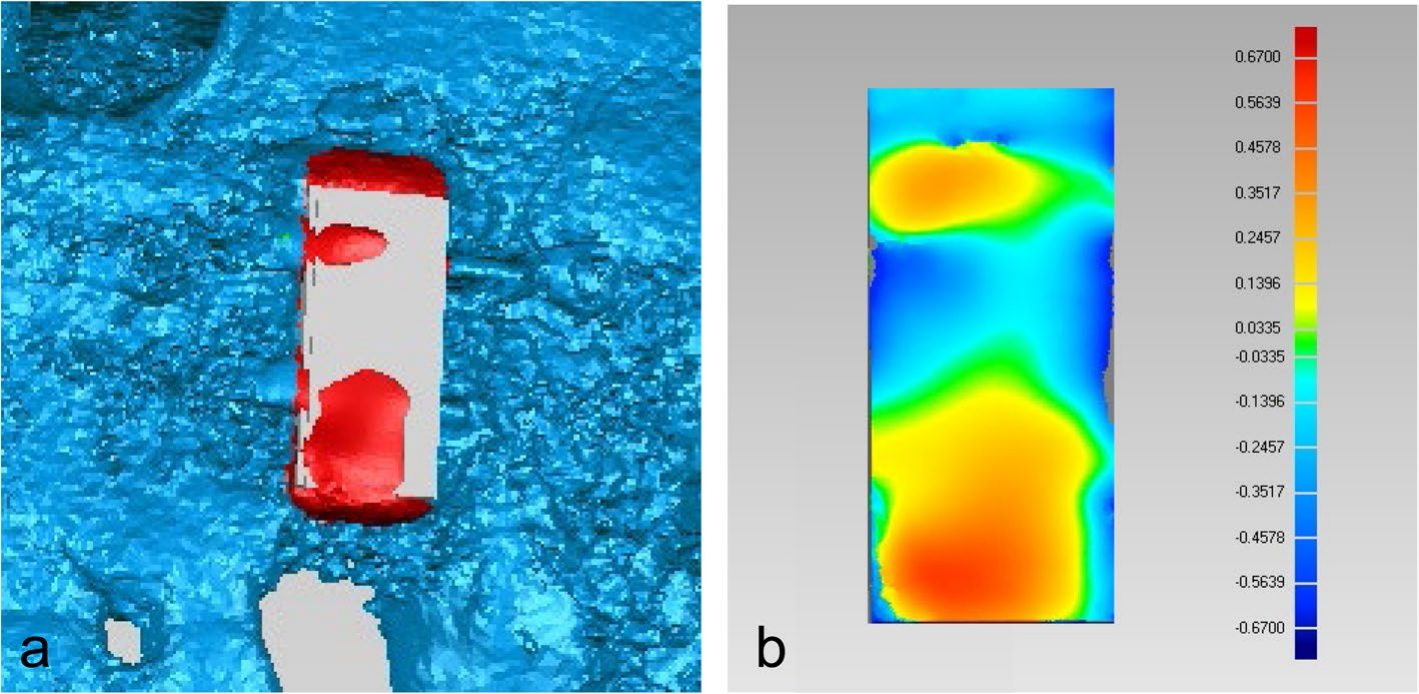
Contour evaluation. **a** the representative image shows the comparison chromatogram between the outer contours of the actual implanted and the planned bone block; **b** the image shows the comparison chromatogram between the two outer contours

Pre- and postoperative jaw models were reconstructed using CBCT. The postoperative jaw models were matched to the preoperative models using "best-fit alignment" in Geomagic software. The outer surface contours of the designed and actual grafts were then selected for 3D deviation analysis. The "Deviation" command produced a 3D color-coded mapping where the difference between the two models was represented by the change in color (Fig. 5). The root mean square estimate (RMSE) of the two models was used to assess the accuracy of the digital template.

#### Horizontal bone gain

At the time of surgery, bone width was measured 1 mm below the highest point of the remaining alveolar bone using a calibrated caliper. The bone width was repeatedly measured during the reentry surgery for implant insertion. Horizontal bone gain was the difference between the bone width before surgery (T0) and before implant placement (T1).

#### Success/survival rate

Bone graft success was assessed based on the following criteria: stable graft during implant placement, absence of pain or discomfort, absence of infection, and absence of bone graft resorption on radiographs [21]. Implant survival was determined according to the following criteria: implant remaining in situ, absence of mobility, and absence of continuous radiolucency on radiographs [22].

#### Peri-implant marginal bone loss

Marginal bone loss was measured on peri-apical X-rays. The peri-implant marginal bone level, i.e., the distance from the implant platform to the most coronal point of bone-implant contact, was measured using ImageJ 1.52a software. The measurement was calibrated with a known distance of the thread pitch. The bone loss around the implant was obtained by comparing the difference in marginal bone level at the follow-up visit with that at the time of prosthesis placement.

#### Surgeon-reported outcome measures

Surgeons were asked to complete a questionnaire to express their evaluation of the digital workflow, including the intuitiveness of the digital process, the ease of understanding the treatment plan, and how helpful the workflow was to their clinical practice according to a previous study [23]. The visual analog scale (VAS) was used to quantify the subjective evaluations. The VAS scores ranged from 0 to 10, in which 0 indicated "very poor" and 10 indicated "very good".

#### Patient-reported outcome measures (PROMs)

Patients were asked to evaluate postoperative sensations of pain, swelling and bleeding via a questionnaire on the day of surgery and at 1, 2, 3 and 7 days postoperatively. The severity of pain, swelling and bleeding is indicated by the patient marking the appropriate scale on a 10 cm visual analog scale, in which 0 indicated "none" and 10 indicated "extreme". The same operator converted the evaluation scores according to the established criteria of the VAS.

### Statistical analysis

Statistical analyses were performed using IBM SPSS Statistics software version 24.0 (IBM Corporation, USA). Descriptive variables that conform to a normal distribution were described by means ± standard deviations (95% confidential interval). Descriptive variables of two groups were compared using independent samples t-test.

## Results

**Fig. 6:**
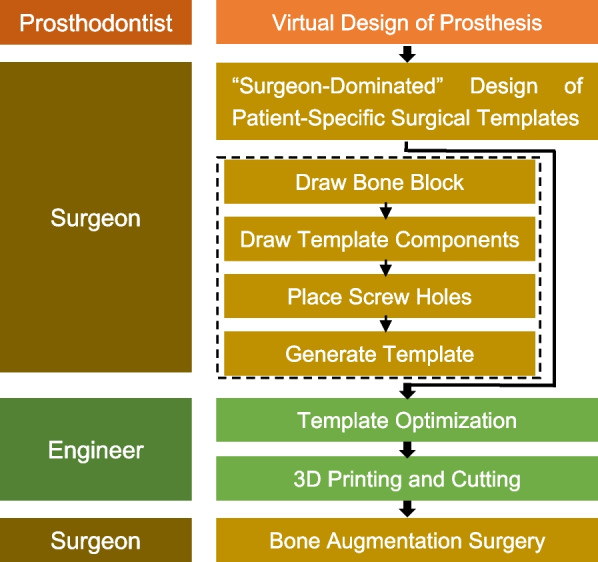
Illustration of digital workflow

The digital workflow for prosthetically oriented onlay bone grafting was developed successfully, and its feasibility was demonstrated. The workflow includes data acquisition, virtual planning, design and manufacture of CAD-CAM surgical templates, model 3D printing, and bone augmentation surgery, which has been presented in a step-by-step manner (Fig. 6). A total of eight patients (5 females and 3 males) with an average age of 37.5 years were enrolled in this study. All patients were followed for at least one year (mean 14.0 ± 1.9 months). The details regarding the patient distribution are shown in Table 1.

**Table 1:** Distribution of the demographic and characteristics

**Patient number**	**Age**	**Gender**	**ASA status**	**Smoking status**	**Defect area**	**Defect size**	**Implant number**	**Implant size**
1	32	Female	I	None	Maxilla	1	1	4.0 × 14 mm
2	35	Female	I	None	Maxilla	1	1	4.0 × 12.5 mm
3	49	Male	II	Smoker	Maxilla	1	1	4.0 × 12.5 mm
4	25	Female	I	None	Mandible	1	1	3.5 × 11 mm
5	45	Male	II	Smoker	Maxilla	1	1	3.5 × 12.5 mm
6	35	Male	I	None	Maxilla	1	1	4.5 × 11 mm
7	38	Female	I	None	Maxilla	1	1	4.0 × 11 mm
8	41	Female	II	None	Mandible	1	1	4.0 × 11 mm

### Bone augmentation data

The bone graft success rate and implant survival rate were 100%. The RMSE values between the preoperative design and the postoperative actual outer contour of bone blocks were 0.41 ± 0.15 (95% CI: 0.28, 0.54) mm, indicating optimal accuracy. There was no significant difference in the RMSE values between the maxilla and mandible. The volumetric change from preoperative to postaugmentation was 378.18 ± 39.04 (95% CI: 345.54, 410.81) mm^3^. The average horizontal bone gain from T0 (bone augmentation surgery) to T1 (implant placement) was 4.79 ± 0.43 (95% CI: 4.43, 5.15) mm. The specific values for each patient are reported in Table 2.

**Table 2:** Horizontal bone gain and RMSE value of each patient

**Patient number**	**Bone width** **(T0) (mm)**	**Bone width** **(T1) (mm)**	**Horizontal bone gain** **(mm)**	**RMSE value** **(mm)**
1	3.5	8.8	5.3	0.46
2	4.0	8.9	4.9	0.20
3	3.2	8.3	5.1	0.58
4	3.1	7.8	4.7	0.32
5	3.0	7.5	4.5	0.60
6	3.7	9.0	5.3	0.25
7	4.2	8.5	4.3	0.36
8	3.8	8.0	4.2	0.51

### Complications

After the bone augmentation procedure, no wound infection, graft mobility, or nerve numbness was observed. However, wound dehiscence occurred in one patient two days after augmentation, resulting in an exposure rate of 12.5%. This complication was treated with 0.12% chlorhexidine mouthwashes and topical application of 1% chlorhexidine gel twice daily. Then, the dehiscence spontaneously reformed the epithelium and gradually healed. Patients had their implants placed 6.1 ± 0.3 months after bone augmentation, as initially planned. At the time of reentry surgery, all the grafts appeared vascularized and well-integrated with the native bone. No complications of bone block separation or fracture occurred during the subsequent implant insertion. The marginal bone loss from T2 (functional loading) to T3 (1-year follow-up) was 0.73 ± 0.32 (95% CI: 0.46, 0.99) mm.

### Surgeon/patient-reported outcomes

According to the evaluation from the surgeons, the digital workflow was advantageous in terms of intuitiveness and helping the surgeons to understand the bone grafting plans. More importantly, the digital workflow helped surgeons with the preoperative design and surgery. Specifically, as shown in Table 3, the average VAS score of digital workflow was 9.3 ± 0.5 (95% CI: 8.7, 9.6) in intuitiveness, 9.0 ± 0.5 (95% CI: 8.6, 9.4) in understanding, and 8.4 ± 1.1 (95% CI: 7.5, 9.3) in helpfulness. Regarding patient-reported outcomes, the patients showed postoperative pain, swelling, and bleeding of varying degrees at different time points in the study (Table 4).

**Table 3:** Mean values and standard deviations (SD) of the VAS scores (0–10) of intuitiveness, ease of understanding and helpfulness of the workflow rated by the surgeons

**Evaluation indicators**	**VAS scores**		
	**Mean ± SD**	**Minimum**	**Maximum**
Intuitiveness	9.3 ± 0.5	9	10
Understanding	9.0 ± 0.5	8	10
Helpfulness	8.4 ± 1.1	7	10

**Table 4:** Mean values and standard deviations (SD) of the VAS scores (0–10) of postoperative sensations of the patients during the different study periods

**Evaluation indicators**	**VAS scores per study period**				
	**5 h**	**1 day**	**2 days**	**3 days**	**7 days**
Pain	3.9 ± 0.6	5.7 ± 0.4	5.8 ± 0.4	5.5 ± 0.3	1.2 ± 0.9
Swelling	1.6 ± 0.5	4.4 ± 0.6	4.7 ± 0.6	5.7 ± 0.7	1.3 ± 1.0
Bleeding	1.6 ± 0.9	1.6 ± 0.3	1.0 ± 0.6	0.4 ± 0.4	0

### Time and economic costs

The time for virtual planning and design of the templates was 60 min. The time for manufacturing the templates was 240 min. The time for surgical operation was approximately 40 to 50 min. The overall costs of the template production were 3000 yuan ($418), including 600 yuan ($84) for the material, and 2400 yuan ($334) for the labor and machine costs.

## Discussion

In the short-term follow-up, the clinical performance of the novel digital workflow appeared to be encouraging. Conventional freehand onlay bone grafting is challenging and time-consuming. Surgeons must outline the future shape of the bone arch based on empirical assessment and check the bone block position based on limited information. The main advantage of the proposed workflow is that it can simplify the surgical procedure and improve the predictability and efficiency of the surgery [24, 25]. The use of digital processes facilitates the transfer of preoperative simulation results to the treatment execution process, thereby reducing the risk of contradiction between the graft and recipient bone. The proposed technology can help determine the shape and dimension of the bone block under the guidance of the CAD-CAM template, which is the main improvement.

The 3D assistance provided by the template is expected to lead to accurate bone augmentation, avoiding insufficient bone volume and secondary bone grafting. This study assessed the agreement between the preoperative design and the actual implanted outer contour of the bone blocks by reconstructing and comparing the two jaw models [26, 27]. The average measured RMSE value was 0.41 mm, and the maximum error was 0.6 mm, which were smaller than the 0.72 mm of conventional surgery reported in another study [15]. The results indicated that using digital templates could improve the predictability and accuracy of onlay bone graft surgery. The patients were operated by two surgeons with 20 years of extensive clinical experience in onlay bone grafting and 5 years of clinical experience in digital processes. The surgeons’ high rating for intuitiveness, understanding, and helpfulness in the questionnaire proves the ease of digital workflow. However, drawing conclusions based solely on a VAS score without a comparative group may indeed lead to bias.

Another benefit of the proposed workflow is prosthetically driven surgical planning [28]. With the use of the template, the desired final prosthesis design and implant position can be planned in conjunction with the surgical planning of the bone block. It is worth mentioning that the authors of the present study have innovatively incorporated intermediate holes for implant placement guidance in the bone graft template. The implant socket preparation can be guided during the implant placement procedure to achieve an ideal implant position. To the best of our knowledge, the integration of the guidance of onlay bone grafting with implant placement using the same template, has been rarely explored in previous reports.

In the past decade, various digitally guided surgical techniques have been created to perform bone augmentation more efficiently and accurately. Stavola et al. described a bone harvest guide that used a tooth positioning component that was integrated with the osteotomy guide [20]. However, as the tooth undercut differs from the bone tissue undercut, this 1-piece design is susceptible to the limitations of the insertion path. In addition, the tooth positioning component cannot be confirmed before surgery, which may hinder the intraoperative use of the guide to achieve accurate positioning. To solve this problem, the template in the present study adopts a split design, dividing the template into three parts. Different parts of the template are attached by screws. The removable design delicately avoids the problem of difficult seating and removal of the template. In this study, the guide was used successfully for all eight patients and there were no seating problems.

Various materials, such as titanium, cobalt-chromium alloy and polymerizing resins, have been used to fabricate surgical templates [29, 30]. Although polymerizing resin is a convenient material that can be used with a desktop stereolithography 3D printer, it may not be suitable for bone graft templates. In terms of strength, polymerizing resin may not withstand the cutting force of the perforating drill pin, while metallic materials have good strength and retention. In addition, nonmetallic materials have a large volume, which increases the flap size and chances of trauma [31]. Therefore, the templates are manufactured with titanium, thereby rendering a smaller flap and a template with a smaller volume and making the procedure more straightforward. However, in terms of cost, titanium templates are more expensive and require longer cutting times. Although this technique improves the accuracy and efficiency of horizontal bone augmentation, the cost-effective ratio needs to be further improved.

Titanium mesh and autogenous bone block are the two main techniques to reconstruct severe bone defects. Titanium mesh provides excellent adaptability and can be shaped to fit various defect sizes and shapes, allowing for customized reconstructions [32–34]. Moreover, titanium mesh offers good stability and rigidity, maintaining the shape and contour of the reconstructed area. Despite good biocompatibility of titanium mesh, there are still risks of mesh exposure and infection, which may affect the osteogenic result. In addition, titanium mesh is not suitable for load-bearing areas or where large forces are applied. In contrast, autogenous bone blocks, typically harvested from the patient's own body, have excellent stability and load-bearing capacity, and lower risks of infection and rejection. Autologous bone contains live bone cells and growth factors that can promote bone healing and regeneration [4, 21]. However, autologous bone blocks also have disadvantages, including the need for a second surgical area, limited availability, and limited shapes and sizes. The present study showed that an average horizontal bone gain of 4.79 mm was obtained after six months, which was similar to the results reported in previous related studies [35], proving that the present method is feasible for obtaining a relatively stable bone augmentation effect.

The present technique uses an autologous block graft covered with a barrier membrane, which protects the block graft against resorption and simplifies the procedure because of the favorable handling of the collagen membrane. In the early twenty-first century, clinicians began to use autologous bone block grafts in combination with deproteinized bovine bone mineral particles (DBBM) and noncross-linked collagen membranes [36]. Ren’s study demonstrated that barrier membrane prevented inward invasion of soft tissue and created an underlying space supporting bone growth [37]. Several clinical studies have shown that autologous bone blocks combined with nonresorbable or resorbable membranes supported by DBBM particles can minimize resorption of the bone graft during healing [38–40]. Chappuis reported a 10-year surface resorption rate of 0.38 mm (7.7%), which also confirmed these results [41]. The patients in this study were given antibiotics and ibuprofen for infection prevention and pain relief. The postoperative pain and swelling were more severe in the first three days after surgery, and then gradually decreased and basically disappeared by the seventh day. Some studies have reported the use of dexamethasone in addition to antibiotics to prevent swelling [32, 42].

The present study has some limitations that need to be addressed. First, the sample size was relatively small, and the follow-up time was short. Second, since it was designed as a pilot study, the main limitation was the lack of a control group. Both RMSE values and surgeon-related outcome measures using the VAS should be compared with a conventional technique in future comparative studies. In the near future, with the development of machine learning and artificial intelligence algorithms, new software with automatic surgical planning functions that can combine different surgical requirements needs to be developed. Hopefully, the present study will accelerate this digitization process and contribute to the new era of “digitization and precision”.

## Conclusions

Within the study’s limitations, the digital workflow of prosthetically oriented onlay bone grafting for horizontal alveolar augmentation provided encouraging results in terms of accuracy and efficacy. Further comparative studies are required to evaluate the outcomes of this workflow.

## Data Availability

All data generated or analysed during this study are available from the corresponding author on reasonable request.

## Abbreviations

3D: 3-Dimensional
RMSE: Root mean square estimate
VAS: Visual analogue scales
